# Cannabis Use and the Development of Depression in Adolescents: Is There an Established Linear Relationship Between the Two?

**DOI:** 10.7759/cureus.27394

**Published:** 2022-07-28

**Authors:** Chantelle T White, Humaira Shamim, Roba Al Shouli, Ahmad B Habbal, Lubna Mohammed

**Affiliations:** 1 Pediatrics, California Institute of Behavioral Neurosciences & Psychology, Fairfield, USA; 2 Dermatology, California Institute of Behavioral Neurosciences & Psychology, Fairfield, USA; 3 Internal Medicine, California Institute of Behavioral Neurosciences & Psychology, Fairfield, USA

**Keywords:** adolescent drug use, marijuana use, adolescent cannabis use, depression, cannabis use disorder, child and adolescent psychiatry

## Abstract

The period of adolescence is a stage during which individuals experience several physical and psychological changes which increase their vulnerabilities to environmental influences. Cannabis exposure is one environmental factor that may put their health at risk. Various research agree that a connection exists between the onset of depression and cannabis use. Cannabis can affect the adolescents’ brain, making them susceptible to depression. Depression is a disorder that manifests in a myriad of ways that can be detrimental to individuals. This problem is not only confined to the individuals themselves, but it is also a societal problem. The relationship that exists between cannabis use and depression is an extremely complex one. This study’s main objective is to review previously conducted research regarding the association between cannabis use and depression in adolescents.

## Introduction and background

There has been an exponential increase in the number of marijuana users throughout the world. The percentage of marijuana users of ages 12 years and older increased from 11% in 2002 to 17.5% in 2019 in the United States [1]. Cannabis refers to the dried leaves, flowers, stems, and seeds from the Cannabis sativa or Cannabis indica plant [2]. It is often used as a recreational drug. Cannabis is used in several ways. These include smoking it, eating it as edibles, baking it into desserts, or vaping it [3].

There is a growing body of literature on the several ill effects of cannabis use on humans. Cannabis use disorder (CUD) is an underappreciated risk of using cannabis that affects 10% of the 193 million cannabis users worldwide [4]. To further perpetuate this problem, the legalization of non-medical cannabis use in some First-World countries may boost the predominance of CUD by making more potent cannabis products more readily accessible at a lower price [4]. One school of thought is that cannabis use causes cannabis dependency syndrome, schizophreniform psychoses, anxiety and depressive disorders, and acute and perhaps chronic cognitive impairment [5]. It may also contribute to structural and functional changes in brain pathways linked to reward, learning, and addiction pathways [5]. The endocrine system, several neurotransmitter pathways, and regions of the brain are involved in depression. The endocannabinoid system (ECS) and the kynurenine pathway (KP) have been firmly associated with this disorder for several years [6].

Adolescents in this literature review are defined as individuals aged between 12 years and 19 years. During the adolescent years, individuals are more susceptible to the development of depression [7]. This is also the period when adolescents tend to experiment with and become introduced to drug use, including marijuana [7]. Cannabis is arguably one of the most used drugs by adolescents in the world [2]. It is progressively perceived as a “soft drug,” as approximately 70% of high school seniors are of the view that regular use is not very dangerous [2]. This is comparable to 20% of adolescents who held this belief in 1990 [2]. This is of special concern as the adolescent brain is vulnerable to the harmful effects of cannabis [8].

Much research has been done to ascertain the impact of cannabis on the human brain; however, most of them have concentrated on adult samples rather than the adolescent population [9]. It is also noted that there are extensive studies available showing the impact that marijuana has on the development of psychosis but not as many have been done to see the effect marijuana has on the development of depression. There is an ongoing debate concerning the link between cannabis use and depression. To better understand the complexity of this issue, it is important to conduct this literature review. 

In this review, each body of work is examined individually to facilitate a discussion on the pertinent findings. The overall aim is to analyze the scholarly sources chosen to answer the formulated question regarding the existence of a linear relationship between cannabis use and depression in adolescents. This enables the identification of the gaps in the body of knowledge on the subject being examined. This review is important in helping to decipher if there is sufficient evidence to support a hypothesis of an established direct linear association between cannabis use and depression in adolescents. Being aware of the characteristics of this relationship is crucial in shedding light on this significant public health issue that warrants the intervention of the pertinent stakeholders.

Search strategy

An extensive review was made on the following databases: PubMed, Google Scholar, Semantic Scholar, and Science Direct. The keywords that were used included; "cannabis, marijuana, hemp, marijuana abuse, depression, sadness, major depression, depressive disorder." Articles were included if they satisfied the following criteria (i) published within the last 12 years, (ii) adolescents 12 years of age to 19 years of age, (iii) published in English, (iv) journal articles, systematic reviews, meta-analyses, clinical trials, observational studies. For this traditional review, the Scale for Assessment of Narrative Review Articles-SANRA was used as a guideline. 

## Review

Discussion

In this century, cannabis use is extremely widespread, especially since it is legalized in many countries, including the United States of America. Since this legalization, the discussion on cannabis use, and its effects on the human body, particularly the brain, has been brought to the forefront of medical research. There is a vibrant ongoing debate pertaining to cannabis use and its association with depression in adolescents. This review will examine the following: the endocannabinoid system, the effects of cannabis use on the brain, the complex relationship between cannabis use and depression, the demographic nexus between cannabis use and depression, and the behavioral factors associated with cannabis use.

The endocannabinoid system

Cannabis - A tall Asian herb is also colloquially referred to as marijuana. Marijuana is the psychoactive dried resinous flower buds and leaves of the female hemp or cannabis plant [10]. The main psychoactive chemical in marijuana is delta-nine-tetrahydrocannabinol (THC) [11]. THC and other chemicals in the marijuana plant are transported from the lungs into the bloodstream to the brain when marijuana is smoked [12]. When this happens, many people experience a sense of euphoria and relaxation; however, some may experience anxiety or fear. This experience is increased for first-time users as well as for persons using marijuana in large amounts or marijuana with a high potency [12].

The endocannabinoid system consists of three components: endocannabinoids molecules, CB1 endocannabinoid receptors, which are mostly found in the central nervous system, and CB2 receptors, which are mostly found in the peripheral nervous system, immune cells, and two main degrading enzymes - fatty acid hydrolase and monoacylglycerol acid lipase [13]. Cannabinoid receptors (CB1) are widely distributed throughout the brain and are found in the hippocampus and prefrontal cortex. They play a role in neurotransmitter release and concentrations across excitatory and inhibitory neural systems [14].

The endocannabinoid system is involved in homeostasis, learning, memory, and excitation and can be modulated via glutaminergic (excitatory) or GABAergic (inhibitory) neurotransmitters [15]. The effects of exogenous cannabinoids are less selective than endogenous cannabinoids and therefore have a wider effect on receptors throughout the nervous system [15]. Anandamide (ANA) is an endocannabinoid made by the body which binds to CB1 receptors, while THC is a phytocannabinoid produced by the cannabis plant. In the endocannabinoid system, there is a recognized ‘lock and key’ theory in which the cannabinoid receptor is the ‘lock’, and the endocannabinoid molecule (ANA for example) represents the key. The endocannabinoid ‘key’ fits/attaches to the cannabinoid receptor ‘lock’ on the cell wall triggering a reaction and release of neurotransmitters resulting in an effect on the brain and body [16]. THC has a similar chemical structure to anandamide, allowing the body to recognize THC as endogenous [12]. THC binds to cannabinoid receptors in the brain, altering the function of the hippocampus, prefrontal cortex, cerebellum, and basal ganglia [12]. As a result, marijuana affects memory, posture, the ability to learn, and reaction time. It affects the brain’s reward system as the THC stimulates neurons, causing the release of dopamine at greater than normal levels, which can lead to the brain becoming more addicted to marijuana [12].

**Figure 1:**
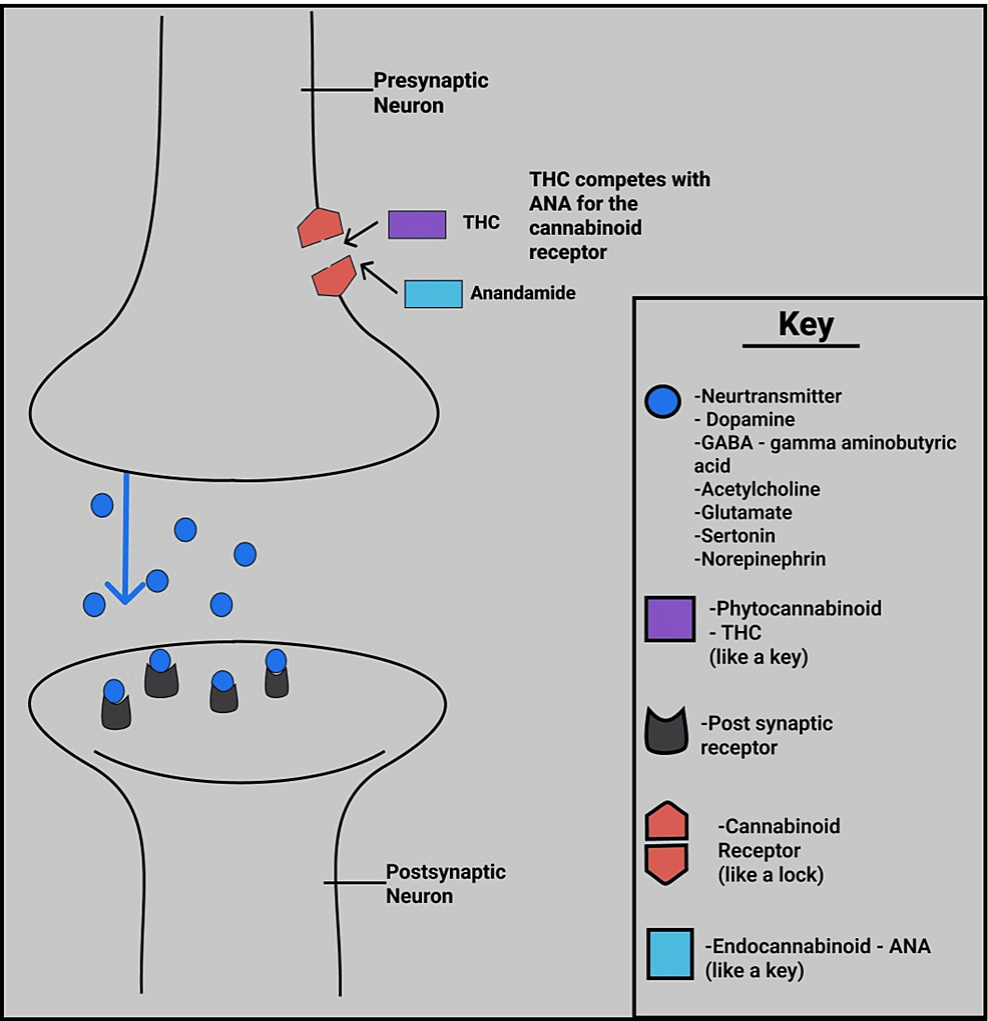
Diagram Depicting the ‘Lock and Key’ System of the Cannabinoid Receptors and Endocannabinoids THC: delta -9-tetra hydrocannabinol, ANA: Anandamide, GABA: gamma aminobutyric acid (Figure created by White C.)

Figure 1 below demonstrates the endocannabinoid system and receptors and how THC competes with ANA, affecting the release of neuroreceptors [17].

Effects of cannabis on the adolescent brain

The adolescent’s brain is still developing and undergoes major remodeling particularly in the prefrontal cortex-an area involved in concentration, higher cognitive function, decision making, planning, and controlling impulses [18]. The prefrontal cortex is involved in our personality and in our ability to have appropriate social responses [19]. The synapses and neurotransmitters of the prefrontal cortex undergo much rearrangement in the adolescent brain as the brain completes maturation to go into adulthood [18]. The fact that there is such a great degree of remodeling occurring in the adolescent brain makes their brains particularly vulnerable to drugs and the effects of cannabis. This is even more compounded by the fact that adolescence marks the time in which persons tend to take chances and experiment with drugs, especially cannabis. In fact, according to the United Nations Office on Drugs and Crime, cannabis is the main drug used among adolescents [20]. On a global scale, 13 million students ages 15-16 years used cannabis in 2018 [20]. In 2018 cannabis accounted for 192 million worldwide users in comparison to cocaine at 19 million, ecstasy at 21 million and amphetamines at 27 million [20]. The number of adolescents in 2019 who initiated marijuana use in the past year averaged to about 3,700 adolescents each day [1]. Additionally, approximately 8.9% of cannabis users transition from use to dependence, with about 4.5% of them doing so within five years [21].

Relatedly, the vulnerability of the adolescent’s brain and the high use of cannabis heightens the concern regarding the effect that this drug has on depression in the adolescent. The World Health Organization (WHO) states that one in seven persons aged 10-19 years faces a mental disorder, and of these, depression and anxiety are among the most prominent causes [22]. It is found that in children aged 10-14 years, depression accounts for 1.1% of emotional disorders, while in adolescents 15-19 years, it accounts for 2.8% [22]. Clearly, there is confirmation that cannabis has a direct effect on the human brain. Correspondingly, adolescents who engage in the use of cannabis may be at risk of developing depression.

The complex relationship between cannabis use and depression in adolescents

According to the Diagnostic and Statistical Manual of Mental Disorders (DSM-V), depressive disorders include disruptive mood dysregulation disorder, major depressive disorder (including major depressive episode), persistent depressive disorder (dysthymia), premenstrual dysphoric disorder, substance/medication-induced depressive disorder, depressive disorder due to another medical condition, other specified depressive disorder [23]. Major depressive disorder involves the presence of a sad, empty, or irritable mood, accompanied by somatic and cognitive changes that substantially affect the individual's capability to function [23]. It is characterized by distinct episodes of at least two weeks duration involving obvious changes in affect, cognition, neurovegetative functions, and inter-episode remissions [23]. Adolescents manifest their depressive symptoms in atypical ways when compared to adults. They may present with poor academic performance, delinquency, irritability rather than sadness, overactivity, aggression or may become socially withdrawn [24]. As cannabis affects the brain chemistry, it may cause depression which manifests as any of these signs. It is, therefore, pertinent to conduct this review to determine the intricacies of the correlation between the use of cannabis and depression.

The relationship that exists between depression and cannabis use may be described as an increasingly complex one. This is because adolescence is a period when teens are usually prone to developing depression due to the various changes in their brain chemistry. Additionally, this is a period when experimentation with drugs is also widespread. The complexity between the use of cannabis and depression is magnified as one would intuitively assume that using cannabis frequently would promote a greater depression rate in adolescents. A longitudinal study reviewed a sample of just under 88,000 participants aged 12-17 years between 2012 and 2017 in which they sought to examine the association between the frequency of cannabis use and major depressive disorder (MDD). The authors described their findings on the regularity of the use of cannabis and the development of depression as contrary to what would be expected. A remarkable finding from this study’s analyses is that cannabis users who used it more often in the previous year had lower rates of depression when compared to those who used cannabis less often in the previous year [7]. This magnifies the debate about the difficulty in determining a direct link between the severity of cannabis use and the severity of depression in adolescents. Their argument is that since adolescents who are cannabis users may be prone to depression, it would be reasonable to assume that there is dose relationship involved [7]. This finding is also supported by other studies. One of these studies include one which involved 178 undergraduate students from Colorado State University in an ongoing research program. Interestingly, it was found that the strongest connection between cannabis use and mood disorder, including depression, was strongest in the groups of persons who used cannabis less frequently or who were casual users [25]. This finding is contradicted in another study-a systematic review and meta-analysis done to explore the association and directionality between mental health disorders and substance use among adolescents and young adults in the U.S. and Canada. This study found that the overall pooled estimate for depression among heavy cannabis users was higher compared to light users [26].

Another controversy surrounding the issue of cannabis use and depression is the existence of two competing hypotheses. These are the cannabis effect hypotheses which imply that cannabis use contributes to depressive symptoms later in life, and the self-medication hypothesis, which justifies that people increase their use of drugs to alleviate their troubling psychological symptoms. One study sought to assess the validity of these hypotheses by exploring associations between the use of cannabis and depressive symptoms in 264 low-socioeconomic-status ‘at risk’ young males. This study posits that the covariation between cannabis use, and depressive symptoms is best explained bidirectionally, with each variable equally worsening the other over a period [27]. Contrastingly, they propose that a common-factors perspective reveals that the relationship between cannabis use, and depressive symptoms may not directly influence each other’s formation rather, it occurs because they share common underlying risk factors [27].

Some authors postulate that the relationship between cannabis and mental health is non-linear. One prospective study focused on the frequency of cannabis use in different age groups and assessed if it caused symptoms of psychosis, depression, and anxiety in a group of Canadian adolescents and adults. The authors propose that there is a possibility that the relationship between cannabis and mental health indicators are non-linear because psychotic and mood disorders may be episodic [28]. They also maintain that the impact of cannabis use and CUD on mental health have been hard to resolve [28]. They reported that persons who were already experiencing depression or psychosis got worse symptoms if they began or continued to use cannabis. On the other hand, if persons abstain from or reduce their use of cannabis, their psychotic symptoms or mood may improve [28]. This nonlinear relationship was also supported by a case series in which 202 American Indian adolescents ages 12-17 years were studied [29]. Three sets of analyses are done to fulfill the aims of the study. The findings of the research highlight that the increased rate of depression symptoms and major depressive episode (MDE) is found among the participants and that cannabis dependence is related to depression symptoms and MDE in boys only and not in girls.

There is also the suggestion of a temporal relationship between substance use and symptoms of depression since cannabis use and depression in both boys and girls co-emerge in late childhood and early adolescence. The study confirms that the change between substance use and depression symptoms occurs cyclically and that they reinforce each other equally [29]. The varying findings from these previous studies accentuate this research’s proposition that it is difficult to explain the characteristics of the link between cannabis use and depression in adolescents. Table 1 below shows various findings from several studies conducted on cannabis use and depression in adolescents. It highlights the complex relationship between cannabis use and depression in adolescents.

**Table 1:** Various Findings From Studies on Cannabis Use and Depression in Adolescents

Author	Type of Study	Summary of Findings
Gilder et al. 2012 [29]	Observational Study	The study included 202 American Indian adolescents, ages 12-17 years. The study suggests that there is a temporal relationship between substance use and depression symptoms since cannabis use and depression in individuals co-emerge in early adolescence. It posits that there is a mutually reinforcing circular evolution between substance use and depression symptoms.
Troup et al. 2016 [25]	Longitudinal Study	This study included 178 Colorado students in ongoing research. It found that the correlation between cannabis use and depression was strongest in those who used cannabis less frequently or were casual users.
Womack et al. 2016 [27]	Longitudinal Study	This study which included 264 ‘at risk’ young men, found that the covariation between cannabis use and depression symptoms is best explained directionally, with each variable worsening each other over sometime. Additionally, there may be no direct influence on the development of each other rather, it may occur because they share common underlying risk factors.
Esmaeelzadeh et al. 2018 [26]	Systematic Review and Meta-analysis	This study explored the association and directionality between mental health disorders and substance abuse in adolescents and young adults in Canada. It found that depression among heavy cannabis users was higher compared to the light users.
Leadbeater et al. 2019 [28]	Prospective Study	The participants in this study were Canadian adolescents and young adults. It found that the relationship between cannabis users and mental health indicators is non-linear because psychotic and mood disorders may be episodic.
Gukasyan et al. 2020 [7]	Longitudinal Study	This longitudinal study was conducted between 2012 & 2017, with just under 88,000 participants ages 12-17 years. It found that adolescents who used cannabis more often in a prior year had lower rates of depression compared to those who used it less often over the previous year.

Demographic nexus between cannabis use and depression

It is difficult to assess if there is a direct linear relationship between the use of cannabis and the progression of depression as there are many compounding variables involved with its use. These include demographic, biological genetic, and behavioral factors. The demographic factors include age, gender, socio-economic status, and educational standing. The behavioral factors comprise a frequency of use, self-medicating, and the overlap of other substance use and misuse-coined ‘polysubstance use.’ Additionally, the relationship is difficult to assess as the potency of cannabis differs depending on how it is consumed, and the exact frequency is found to be difficult to determine [30].

It is found that age puts adolescents at risk of using cannabis and developing depression. In one cross-sectional study which examines over 36,000 Norwegian adolescents ages 13-17 years, it is found that cannabis use and depression frequency increases with age [31]. Similar findings are noted in another cohort study that looks at the frequency of cannabis use and depressive symptoms and suicidal ideation in 1,606 Canadian youths 15-20 years of age. It is found that younger adolescents are less inclined to use cannabis: 7% at age 15 years, but as they get older were more likely to use this drug: 16% by age 20 years, and that once they begin using, they are 11-15 times more likely to continue use into their lifetime [30].

Another risk factor for cannabis use is gender. It is found that males are more prone to using cannabis than females [30,31]. Interestingly, although males use the drug more, females are more likely to develop depression when they use cannabis [30,31]. This finding is contradicted by other studies, which found that cannabis dependence is associated with depression symptoms and major depressive episodes in boys only and not in girls [29]. The biological effects of cannabis may lead to the later development of CUD and MDD [29]. It should also be noted that cannabis use and later depressive symptoms may be caused by other genetic and/or genetical risk factors [27,32]. It is vital to mention that though many studies confirm a positive relationship between the use of cannabis and depression in adolescents, some researches are unable to identify this association [33]. One systematic review found that because cannabis use effects motivation and depression are usually manifested at the highest levels of use, it is difficult to deduce if problems with motivation or depression are specifically because of cannabis use [9].

**Figure 2:**
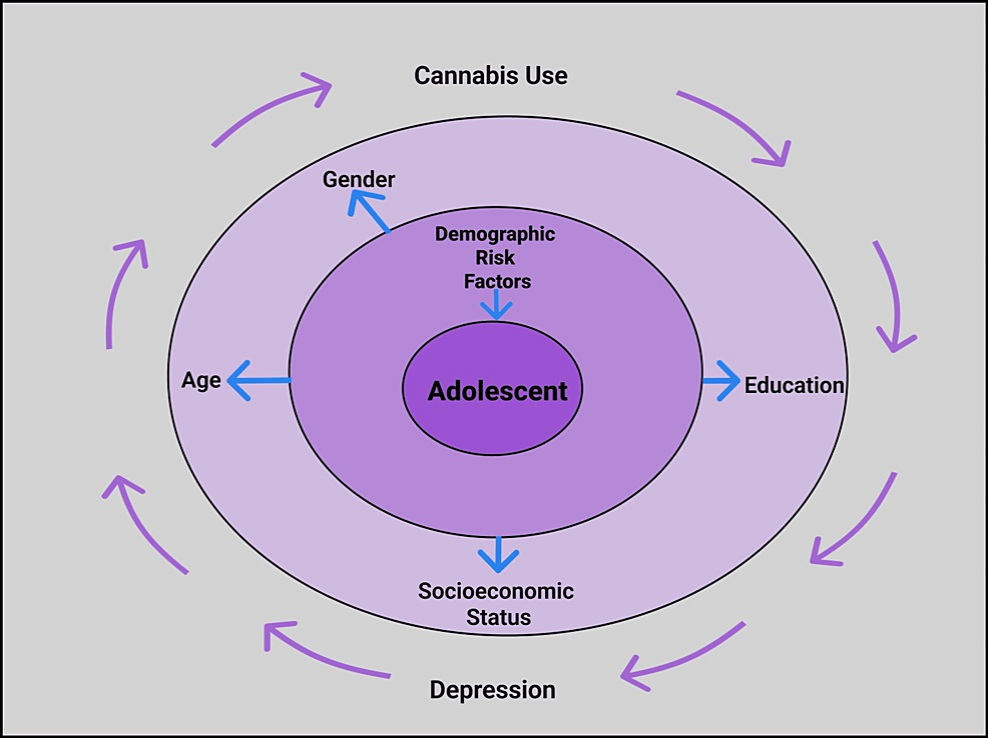
Demographic Risk Factors and the Cyclical Relationship between Depression and Cannabis Use in adolescents. (Figure created by White C.)

Another significant factor that impacts the relationship between cannabis use and depression is the socioeconomic status (SES) of adolescents. Potential third variables may underlie the contemporaneous covariation between cannabis use and depressive symptoms (common-factors perspective); however, the authors found that after accounting for many covariates such as income and education, the association between cannabis use and depression was reduced [27]. Another study found the opposite effect, where after adjusting for family SES the correlation between cannabis use and depression remained the same [30]. A combination of these factors causes the complexity in explaining if there is a direct linear relationship between cannabis use and depression in adolescents. Figure 2 below depicts the various demographic risk factors impacting adolescents and the cyclical relationship between cannabis use and depression.

Behavioral factors associated with cannabis use

Several behavioral factors impact adolescent cannabis use. These include the frequency of use, the way it is used, poly-substance use, self-medicating habits, and the potency of cannabis. This makes it more complicated to establish if there is a direct correlation between the use of cannabis and the development of depression, as potency and frequency can be challenging to measure. The potency of THC is higher in cannabis formulated as wax, dabs, edibles, and that used in a vape, and these are increasingly used by adolescents in the United States of America [34]. The association between cannabis usage and symptomatology of anxiety and mood disorder is complicated since, despite a myriad of research on the topic, confirmation of this relationship remains ambiguous. The pattern of use of cannabis, the composition-synthetic or natural, and the cultural backgrounds of the users are important factors that further complicate the issue [25].

According to two studies, it was impossible to evaluate the exact amount of cannabis that the adolescent consumed since it is the frequency of use that was measured and not the quantity [35,9]. ‘Poly-substance’ use is quite prevalent in teens. Cannabis users often use other drugs as well, such as alcohol, tobacco cocaine, hallucinogens, and amphetamines [30]. This makes it difficult to ascertain if it is the substance use alone or if it is the combination with cannabis that contribute to depression development.

**Figure 3:**
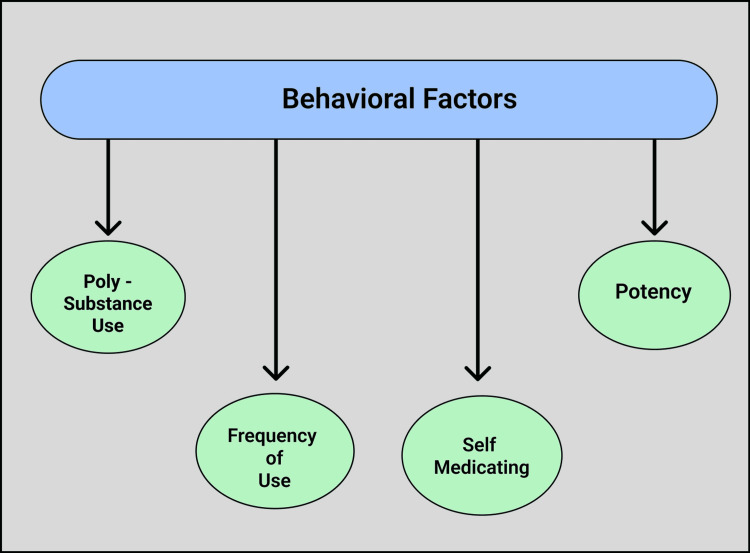
Behavioral Factors Associated With Cannabis Use (Figure created by White C.)

Self-medicating is another factor that complicates the determination of the direct relationship between cannabis use and depression in adolescents. The substance use hypothesis, which is supported in one study, speculated that persons with symptoms of depression or anxiety use cannabis to relieve mental issues [31]. It is also found that adolescents who had depression at the age of 15 years increase their risk of using cannabis weekly by the age of 20 years, thus supporting this hypothesis [30]. These varied elements complicate the characteristics of the link that exists between cannabis use and depression in adolescents. Figure 3 below depicts some behavioral factors that perpetuate the complexity of the relationship between cannabis use and depression in adolescents.

All these factors highlight the complexity of the relationship between cannabis use and depression in adolescents. As observed, the demographic nexus between cannabis use and depression in adolescents is informed by specific factors. Additionally, there are behavioral factors that impact the association in a myriad of ways.

Limitations

Although this study has successfully demonstrated that due to a strong combination of dependent factors, there is much complexity concerning the relationship between cannabis use and depression, it has certain limitations. The chief limitation is that some of the studies reviewed used different measurements for depression and cannabis use. The sample sizes varied significantly across the studies, therefore making it difficult to generalize data. Another shortcoming is that some of the studies used self-reports, thus making the findings susceptible to over or under-reporting and errors.

## Conclusions

This study presents important insights into the relationship between cannabis use and depression, with a focus on the linear relationship between the two. The studies reviewed provide considerable evidence of a strong relationship between cannabis use and depression in adolescents. Findings from these studies also show evidence of some compounding factors that impact the use of cannabis and the development of depression in adolescents. This makes it even more difficult to evaluate if there is a direct linear relationship between cannabis use and depression. Therefore, it can be inferred that there is an indistinct correlation between cannabis use and depression in adolescents. The importance of this research is that our findings contribute to the existing knowledge about cannabis use and depression and identify the factors which make it difficult to determine the cause/effect paradox of cannabis use and depression. This research will help to guide approaches that can be considered as well as encourage further investigation on this topic.

We recommend that future research on cannabis use and depression focus on more prospective studies in which subjects are followed over longer periods and have larger sample sizes. It is also suggested that more animal studies should be conducted to see the effect of specific doses of cannabis which may lead to depression, to better clarify the potency issue. Additionally, future researchers should control for polysubstance use to better identify the effect of cannabis only. If this gray area concerning the linear relationship between cannabis use and depression is further investigated, this would be very impactful in demystifying this issue. This would impact treatment options and enhance more targeted prevention methods.
